# Watch the GAP: Emerging Roles for IQ Motif-Containing GTPase-Activating Proteins IQGAPs in Hepatocellular Carcinoma

**DOI:** 10.1155/2012/958673

**Published:** 2012-09-03

**Authors:** Valentina A. Schmidt

**Affiliations:** ^1^Department of Medicine, Stony Brook University, Stony Brook, NY 11794-8151, USA; ^2^Division of Hematology, Stony Brook University, HSC T15-053, Stony Brook, NY 11794-8151, USA

## Abstract

IQ motif-containing GTPase-activating proteins IQGAP1 and IQGAP2 are highly homologous multidomain scaffolding proteins. Their major function consists of integration of Rho GTPase and Ca^2+^/calmodulin signals with cell adhesive and cytoskeletal reorganizational events. Recent studies showed that they play an important role in carcinogenesis. There is growing evidence that IQGAP2 is a novel tumor suppressor counteracting the effects of IQGAP1, an oncogene, in several cancers, especially in hepatocellular carcinoma (HCC). While HCC is highly prevalent and one of the deadliest cancers worldwide, the signaling pathways involved are not fully understood and treatment of advanced disease still represents an area of high unmet medical need. This paper compiles various findings from studies in mouse models, cell lines, and patient samples that support future development of IQGAPs into new therapeutic targets. It also discusses distinct features of IQGAP2 in an attempt to provide insight into the mechanism of the seemingly paradoxical opposing roles of the two very similar IQGAP proteins in carcinogenesis.

## 1. Introduction

 This paper focuses on the recently recognized role of IQ motif-containing GTPase-activating proteins (IQGAPs) in hepatic carcinogenesis. Hepatocellular carcinoma (HCC), the most common form of primary liver cancer [1], is the second and the sixth leading cause of cancer-related death worldwide in men and women, respectively [2]. HCC is responsible for between 500,000 and 1 million worldwide deaths annually [3]. HCC etiologies are diverse and include chronic hepatitis B (HBV) and C (HCV), chronic excessive alcohol consumption, steatosis, diabetes, and exposure to toxic agents such as aflatoxin B1, or any hepatic disease associated with cirrhosis. While the highest HCC rates are found in East and South East Asia, HCC incidence is increasing in many parts of the world, including the United States, most probably due to the rising incidence of HCV infection [4]. In the US, HCC incidence rates tripled between 1975 and 2005 [1]. In 2005, HCC incidence was estimated at 667,000 cases globally and 17,550 cases in the United States [5]. Overall survival of patients with HCC has not improved in the last two decades. Since chronic liver disease can often be asymptomatic in early stages, the majority of HCC patients are diagnosed late in the course of their disease and the five-year survival rate in such patients is only 5% [6]. While the only significant improvement in overall survival for patients with advanced HCC was reported for the multikinase inhibitor sorafenib [7], the clinical benefit derived with sorafenib occurs mainly through disease stabilization and curative agents for advanced HCC are yet to be developed.

 The diverse etiology of HCC infers a considerable genomic heterogeneity and involvement of multiple signaling pathways in the disease pathogenesis. The heterogeneity and instability of human tumors pose a serious impediment to identification of target genes for cancer therapy, making genetically well-defined mouse models increasingly important in cancer research. A multitude of molecules has been identified as potential therapeutic targets for HCC and described in many excellent reviews [8–10]. Here, we survey involvement of a significantly understudied protein, IQGAP2, and its homolog, IQGAP1, in cancer with more specific emphasis on the development of HCC. Compelling recent data have introduced IQGAP2 as the newest addition to the long list of HCC-related tumor suppressors and potential molecular targets for much needed curative therapy.

## 2. Multifunctional IQGAP Protein Family

**Figure 1:**
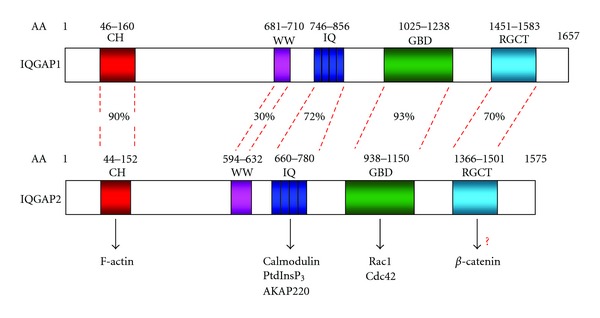
The domain structure of human IQGAP1 and IQGAP2. CH (calponin homology) domain, WW, polyproline binding domain, IQ calmodulin-binding motif, GBD-GTPase binding domain, and RGCT-RasGAP C-terminus domain. Domain percent homology is shown. Adapted from [15, 16]. Also shown binding partners of IQGAP2 identified to date. While it has been confirmed that IQGAP2 co-immunoprecipitates with *β*-catenin [27], RGCT is marked as a domain responsible for *β*-catenin binding based on analogy with IQGAP1. A list of IQGAP1 numerous binding partners can be found in [28].

 IQGAPs represent a small subgroup of an evolutionally conserved superfamily of GTPase-activating proteins (GAPs) [11]. In humans and mice, the IQGAP protein family consists of three members-IQGAP1, IQGAP2 and IQGAP3 [12–15]. IQGAPs are highly homologous multidomain proteins that integrate Rho GTPase and Ca^2+^/calmodulin signals with cell adhesive and cytoskeletal reorganizational events [16]. All three are large cytoplasmic scaffolding proteins (MW 180–190 kDa). Their domain structure includes an actin-binding calponin homology (CH) domain, a single WW domain capable of binding various proline-rich proteins, four IQ motifs binding calmodulin, a large GTPase binding domain (GBD) known to bind Rho GTPases Rac1 and cdc42, and a RasGAP C-terminus domain (RGCT) (Figure 1) [16]. The GTPase binding domain in IQGAPs lacks an arginine residue, essential for GTP-hydrolysis, which may explain why none of the IQGAPs demonstrated *in vitro* GAP activity toward GTPases [17]. Rather, IQGAPs are believed to stabilize the active GTP-bound form of both Rac1 and cdc42 [18, 19]. Among the three homologs, IQGAP1 is the most extensively studied to date. IQGAP1 has been shown to play a role in multiple cellular processes requiring cytoskeletal rearrangement, including cell motility, polarity, proliferation, and differentiation [16]. Significant amount of evidence also implicates IQGAP1 in promoting tumorigenesis in various cancers [20, 21], characterizing it as a *bona fide* oncogene. Unlike its ubiquitously expressed homolog, IQGAP2 is predominantly expressed in liver, kidney and platelets [13, 22], and its physiological role remains largely understudied. Both IQGAP1 and IQGAP2 appear to have functional significance in platelets. IQGAP1 may modulate platelet procoagulant function by regulating the secretory pathway of *α*-granule exocytosis [23], while IQGAP2 functions as a unique scaffolding protein linking thrombin activation to platelet cytoskeletal actin assembly and reorganization, a finding consistent with the IQGAP2 domain structure [22]. Peculiar chromosomal localization of the IQGAP2 gene within the proteinase activated receptor (PAR) gene cluster in both the human and mouse genome [22, 24] also supports the notion that IQGAP2 and PARs may be components of a functional genomic unit uniquely evolved to facilitate thrombin-mediated signaling. The most recently discovered member of the IQGAP family, IQGAP3, is expressed predominantly in the brain and regulates neurite outgrowth [15]. There also have been two reports on potential function of IQGAP3 in liver. A genome-wide microarray expression profiling of 24 HCC tumors revealed upregulation of the IQGAP3 gene, along with several other genes located at 1q22, a region commonly amplified in HCC [25]. IQGAP3 expression was also shown to be increased in proliferating hepatocytes in a mouse liver regeneration model [26].

## 3. IQGAP1 and IQGAP2 Play Opposing Roles in Hepatic Carcinogenesis in Mice

 While the molecular mechanisms leading to HCC may differ by etiology, it generally evolves through a multistep process involving hepatocyte destruction, proliferation and regeneration. At the molecular level, both genetic and epigenetic alterations have been observed in HCC tumors that result in abnormal expression of genes involved in cell cycle control, cell growth and proliferation, apoptosis, and cell-cell interactions [29]. Recurrent allelic losses or gains have been detected on 14 chromosome arms in more than 30% of all HCC analyzed [29]. Notably, 5q13, the region of localization of IQGAP2 gene, is not found among them. Similarly, IQGAPs were not among proteins implicated in the development of HCV-related HCC based on an expansive gene profiling study [30]. Still, given the heterogeneity of this disease, it is highly unlikely that all genes involved have been identified to date. Unlike IQGAP1, IQGAP2 has never been implicated in carcinogenesis until now.

 Generation of a conventional knockout mouse lacking the *Iqgap2 *gene [27] provided a first insight into the physiological functions of IQGAP2. *Iqgap2* ^−/−^ mice demonstrated an age-dependent increase in apoptosis and a structural mitochondrial defect in hepatocytes [27]. Furthermore, 86% of *Iqgap2* ^−/−^ mice (*N* = 18/21) developed HCC by the age of 18–24 months [27]. Both sexes were affected equally and no other malignancies were evident. At the molecular level, *Iqgap2* ^−/−^ HCC tumors were characterized by an 8-fold increase in cyclin D1 levels (a *β*-catenin nuclear target), a 9-fold upregulation of cytoplasmic IQGAP1 expression and loss of membrane E-cadherin expression. HCC development in *Iqgap2* ^−/−^ mice was linked to the Wnt-*β*-catenin pathway activation, supported by, in addition to cyclin D1 upregulation, *β*-catenin translocation from the cellular membrane and accumulation of its dephosphorylated (active) form. Interestingly, more moderate (*∼*2.5-fold) increase in cyclin D1 and IQGAP1 expression in the livers of younger *Iqgap2* ^−/−^ mice without HCC was also evident, establishing that IQGAP1 upregulation and cyclin D1 activation predated histological evidence for HCC development in these mice [27]. An elevated serum level of aspartate aminotransferase (AST) was detected in *Iqgap2* ^−/−^ mice as early as 4 months of age. Late onset of HCC development in *Iqgap2* ^−/−^ mice was consistent with the timeframe of human disease, which undergoes multiple phases and often takes decades to progress to carcinoma. While IQGAP2 was found in abundance in platelets [22], *Iqgap2* ^−/−^ mice displayed platelet function indistinguishable from the wild-type littermates, probably due to a certain functional redundancy of IQGAPs in platelet activation.

**Figure 2:**
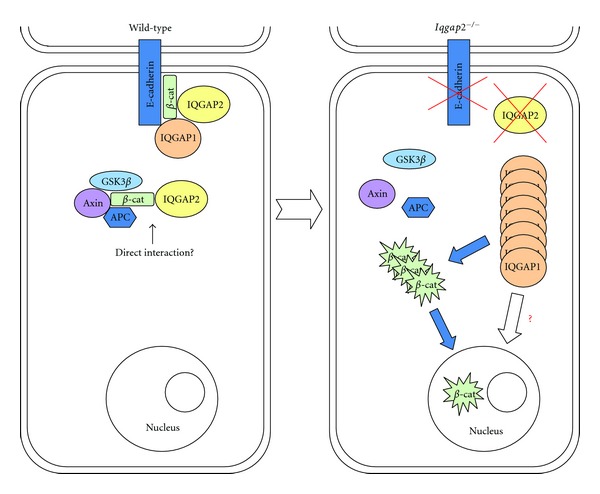
Schema summarizing the proposed hypothesis for IQGAP1 and IQGAP2 involvement in HCC development. In wild-type hepatocytes, IQGAP2 exists in two pools-bound to *β*-catenin and anchored at the submembrane region along with E-cadherin and IQGAP1, and as a part of the *β*-catenin destruction complex consisting of GSK3*β* kinase, Axin and Adenomatous polyposis coli (APC). The *β*-catenin destruction complex prevents *β*-catenin activation and translocation to the nucleus. In *Iqgap2* ^−/−^ hepatocytes, E-cadherin disappears from the membrane, while *β*-catenin escapes the destruction complex, accumulates in the cytoplasm, and enters the nucleus, where it initiates transcription of various target genes. Simultaneously, IQGAP1, released from the submembrane region, is upregulated and perhaps acts in the similar to active *β*-catenin manner. Overexpressed IQGAP1 may also stimulate activity of destabilized *β*-catenin.

 Even more surprisingly, when *Iqgap2* ^−/−^ mice were crossed with *Iqgap1* ^−/−^ mice [31], the resultant *Iqgap1* ^−/−^ */Iqgap2* ^−/−^ mice showed a lower incidence and smaller size of HCC tumors, and improvement of overall survival compared to *Iqgap2* ^−/−^ mice [27]. These data suggest that inactivation of IQGAP1 in mouse liver impairs tumorigenesis caused by IQGAP2 deficiency. According to a proposed model, IQGAP2 plays the role of a tumor suppressor by being a part of the APC/AXIN/GSK3*β* complex, binding cytoplasmic *β*-catenin and preventing it from dephosphorylation and, subsequently, from activating *β*-catenin's nuclear target genes (Figure 2). Data on HCC development in *Iqgap1* ^−/−^ */ Iqgap2* ^−/−^ mice suggest that its mechanism is also IQGAP1-dependent, and, at least in the mouse model, IQGAP1 antagonizes IQGAP2 in liver carcinogenesis. The fact that *Iqgap1* ^−/−^/*Iqgap2* ^−/−^ mice develop HCC, albeit at a lower rate, shows that in the absence of IQGAP2, destabilization of the E-cadherin/*β*-catenin axis is a primary cause of HCC. Noteworthy, IQGAP2 appears to be critical for maintaining cell adhesion during embryonic development of *Xenopus*, where silencing of IQGAP2 resulted in a loss of *β*-catenin and IQGAP1 from cell borders in the ectoderm [32]. Unattended downstream effects of upregulated IQGAP1 in liver may have the role of a “second hit”, exacerbating the HCC development. IQGAP1 has been shown to translocate into the nucleus in late G1/early S phase of the cell cycle and is believed to stimulate DNA replication and progression of the cell cycle [33]. Therefore, it is possible that the observed overexpression of IQGAP1 in *Iqgap2* ^−/−^ livers may result in activation of currently unidentified nuclear oncogenic targets of IQGAP1. Yet, it remains unestablished whether isolated IQGAP1 overexpression is sufficient for HCC development. A study in a rat model of oxidative stress-induced hepatotumorigenesis showed a significant stepwise IQGAP1 upregulation at both the transcript and protein levels throughout tumor progression [34]. Interestingly, in this model IQGAP1 upregulation was tightly linked to increased levels of vimentin, a cytoplasmic intermediate filament protein synthesized in cells of mesenchymal origin, and these findings were confirmed by microarray data mining in human HCC tumors [34]. 

 Alternatively, overexpressed IQGAP1 may realize its oncogenic effect in HCC liver by stimulating *β*-catenin transcriptional activity in the nucleus, which has been described earlier in colon cancer cells [35]. The observation that IQGAP1 has the ability to bind RNA directly [36] supports this hypothesis. It is also known that at the membrane, IQGAP1 competes with *α*-catenin for binding to E-cadherin [37], and destabilization of the *β*-catenin-E-cadherin membrane complex may be another consequence of the IQGAP1 overexpression in IQGAP2-deficiency (Figure 2). Of note, targeted disruption of the murine *Iqgap1* gene caused no defect except for a late onset of gastric mucosal hyperplasia [31]. Given the IQGAP2 multidomain structure, it is feasible to postulate that IQGAP2 may contribute to hepatic carcinogenesis via several mutually nonexclusive and possibly converging mechanisms, which are likely to be identified in the future.

## 4. IQGAP2 Is Silenced in Human Hepatic, Gastric, and Prostate Cancers

 As of now, genetic alterations in the IQGAP2 gene have not been identified in patients, and no human condition has been definitively linked to the gene. Still, single nucleotide polymorphism (SNP) research has recently started to yield evidence for IQGAP2 involvement in certain diseases. A recent genome-wide SNP study identified several SNPs in the human IQGAP2 gene associated with insulin-dependent and noninsulin-dependent type 2 diabetes mellitus [38], emphasizing the significance of exploring the IQGAP2-insulin resistance link. A study in *Iqgap2* ^−/−^ mice supports this notion by showing that these mice are hypoglycemic and protected from diet-induced hepatic steatosis and insulin resistance [39]. Also, the presence of the rs153317CT/TT IQGAP2 genetic variant was linked to a 2-fold increased risk of shorter survival in patients with pancreatic cancer receiving radiation treatment [40]. IQGAP2 SNP rs457717C/T was correlated with age-dependent hearing impairment in yet another genome-wide association study [41]. Finally, IQGAP2 SNP rs3797418 was shown to influence sensitivity of 174 patient lymphoblastoid cell lines to the cytidine analogues gemcitabine and arabinosylcytosin [42]. This is an important finding since gemcitabine is used to treat many solid tumors, including pancreatic, and this SNP might be developed into a predictive marker for drug response.

 Immunohistochemical analysis of a cohort of HCC patient liver specimens showed that IQGAP2 protein expression was reduced in 78% of the samples (64/82) and, consistent with the *Iqgap2* ^−/−^ mouse model, the IQGAP1 protein was overexpressed in 84% of tumors studied [43], thus confirming the relevance of the *Iqgap2* ^−/−^ mouse model to human disease. Both liver samples from normal donors and from patients with cirrhosis showed the reverse trend, suggesting that a hepatic IQGAP1 to IQGAP2 ratio may be developed into a promising biomarker for accurate HCC diagnosis. IQGAP1 protein overexpression in 58% of HCC patient tumors (32/55) compared to normal adjacent tissue was confirmed in another study [44]. A microarray analysis also showed upregulation of IQGAP1 and downregulation of IQGAP2 in late-stage HCC tumors [45]. Lastly, subcellular tissue proteomics analysis of HCC patient tumors revealed a 7.2-fold decrease in IQGAP2 expression and validated IQGAP2 as one among 21 novel candidate molecular targets of HCC [46]. Further studies into a possible epigenetic cause of IQGAP2 silencing in human HCC ruled out promoter hypermethylation as a potential regulatory mechanism of IQGAP2 expression [43]. This is somewhat surprising, since IQGAP2 promoter hypermethylation was identified as a cause of IQGAP2 silencing in gastrointestinal cancers [47]. Additionally, IQGAP2 was shown to be downregulated in gastric cancer by DNA copy number loss [48]. 

 A vast number of recent publications showed that microRNAs (miRNAs), a class of small noncoding RNAs, are involved in posttranscriptional negative regulation of both oncogenes and tumor suppressors in multiple cancers, including HCC [49–51]. Various miRNAs have been implicated in different types of liver disease [52]. In the past several years, miRNAs have been found to be frequently deregulated in HCC, capable of being both oncogenes and tumor suppressors during tumor development and progression [53, 54]. A link between miRNAs and the clinicopathological features of HCC tumors has also been recently established. The most frequently deregulated miRNAs in HCC include the let-7 family (downregulated), miR-122 (downregulated), and miR-221/222 (upregulated) [52, 53]. Of note, miR-122 represents 70% of the total hepatic miRNA population. miRNA expression profiling has also been shown to be useful for classification of HCC molecular subclasses [55]. While the knowledge of how, in turn, miRNA expression is controlled in cancer remains limited, many HCC-related miRNAs have been shown to be silenced as a result of CpG hypermethylation, whereas others, such as miR-151, are overexpressed in HCC because of a chromosomal region gain (8q24 in case of miR-151) [53]. It has been reported recently that miR-124, and miR-203 may regulate *iqgap1* expression in HCC [56]. This is the first report describing regulation of an IQGAP family member by miRNA. MiR-124 and miR-203 were identified as epigenetically silenced in HCC as a result of assessment of the methylation status of 43 loci containing CpG islands around 39 mature miRNA genes in a panel of HCC cell lines and noncancerous liver tissues as controls. Overexpression of miR-124 and miR-203 suppressed HCC cell growth *in vitro*. The 3′-untranslated region (3′-UTR) of IQGAP1 gene was identified as a direct target for both miRNAs, along with cycline-dependent kinase 6, vimentin, and several other proteins. This study characterized miR-124 and miR-203 as novel tumor-suppressive miRNAs in HCC and may provide an explanation for the observed upregulation of IQGAP1 in human HCC tumors [43]. Since IQGAP genes are highly homologous, screening for IQGAP2-specific HCC-relevant miRNAs is well justified and may provide new therapeutic targets. In 2009, an AAV-mediated hepatic delivery of a tumor-suppressing miR-26a proved to be effective in treatment of HCC in a mouse model [57]. 

 Recently, the tumor suppressing repertoire of IQGAP2 has been expanded to include prostate cancer [58]. IQGAP2 was shown to be expressed at significantly reduced levels in tumor specimens from patients with both advanced and androgen-independent prostate cancers. Ectopic overexpression of IQGAP2 reduced proliferation of both DU145 and PC3 prostate cancer cell lines, as well as invasiveness of DU145 cells. This was linked to inhibition of Akt kinase activation [58]. 

## 5. Diverse Binding Partners May Explain How IQGAP2 Counteracts the Effects of IQGAP1 

 While IQGAP1 and IQGAP2 proteins share a domain structure and possess significant sequence homology, they appear to have opposing functions *in vivo*, at the very least in the pathogenesis of cancer. Such an apparent paradoxical phenomenon can be attributed to their distinct tissue expression, subcellular localization and protein binding partners [20], and dissecting the mechanisms underlying the divergent functions of IQGAPs could lead to developing these scaffolding proteins into novel molecular targets for HCC. Other precedents of homologous proteins counteracting each other have been described. For instance, structurally similar heparin-degrading endosulfatases sulfatase 1 (SULF1) and sulfatase 2 (SULF2) have been reported recently as having a role of a tumor suppressor and oncogene, respectively, in HCC by regulating different signaling pathways [59]. Moreover, IQGAP1 levels of expression positively correlated with SULF2 levels in HCC specimens, while IQGAP2 showed negative correlation with SULF2 [59]. SULFs might be regulating the Wnt/*β*-catenin pathway and epithelial-mesenchymal transition (EMT), and functional relationships between IQGAPs and SULFs should be further investigated. 

 An impressive list of confirmed IQGAP1 binding partners has been growing continuously in the past decade. The most recent reviews on the topic [28, 60] list over 40 proteins associated with IQGAP1. Less is known for IQGAP2. Among the proteins associated with IQGAP2 are actin, calmodulin, Rac1, and cdc42 GTPases [20], and also the arp2/3 complex in platelets [22], *β*-catenin in liver [27], protein kinase A-anchoring protein AKAP220 [61] and, most recently identified, phospholipid PtdInsP_3_ [62] (Figure 1). While both IQGAP1 and IQGAP2 bind Rac1 and cdc42, it appears that IQGAP1 selectively binds an inactive, GDP-bound form of these GTPases [18, 63], while IQGAP2 does not discriminate between their GTP- and GDP-bound forms [13, 64]. This is of particular relevance to HCC, because targeted ablation of cdc42 in mouse hepatocytes and bile ducts resulted in the development of HCC, closely resembling the cancer in *Iqgap2* ^−/−^ mice in terms of late onset and molecular signature of the tumors [65]. It was also reported recently that different IQ-motifs in different IQGAPs display selectivity for calmodulin and related proteins, such as myosin essential light chain and S100B [66].

 Another point of IQGAPs divergence that may translate into functional differences is IQGAPs phosphorylation by distinct kinases. It has been reported that phosphorylation of IQGAP1 at Ser1443 by protein kinase C*ε* (PKC*ε*) increases IQGAP1 binding to nucleotide-free Cdc42, leading to the loss of cell-cell contacts [67]. Later, it was shown that phosphorylation controls IQGAP1 switching between pro-growth and pro-cell division/migration conformations, and that failure to switch between them leads to uncontrolled cell proliferation and transformation [68]. While three-dimensional structure is not currently available for either full-length IQGAP, it is anticipated that these proteins possess considerable capacity for conformational change, enabling integration and processing of diverse signals [69]. Most recently, phosphorylation of IQGAP2 has been confirmed [61]. It occurs at Thr716 by cAMP-dependent protein kinase (PKA) and was shown to enhance IQGAP2 binding to the active, GTP-bound form of Rac1 [61]. PKC*ε* and PKA kinases regulate distinct signaling pathways, and while both have a role in cancer progression, their dissimilar activators and targets may also hold a key to deciphering the mechanisms behind the opposing functions of IQGAP1 and IQGAP2 in HCC. PKC*ε* kinase belongs to the so-called novel, calcium-independent, PKCs as opposed to the classical calcium-dependent PKC group, which includes PKC*α*, *β*, and *γ*. All PKCs are major targets of tumor promoting phorbol esters and diacylglycerol (DAG) in response to the activation of growth factor receptors [70]. PKC*ε* has been described as an oncogene and a tumor biomarker in many cancers, and it realizes its oncogenic effects mostly through the modulation of the Ras signaling cascade, phosphorylation of the Bcl-2 family proteins and activation of the Akt signaling pathway [71]. PKC*ε*, in turn, is regulated by the phosphoinositide-dependent kinase 1 (PDK1) and also by autophosphorylation [72]. PKA kinase modulates cell adhesion-related events, including migration, along with glucose and lipid metabolism [73]. It is regulated by integrin-mediated cell adhesion to extracellular matrix (ECM). Molecular targets of PKA are numerous and include actin, *α*4 integrins, VASP, Rho GTPases, p21-activated kinase-1 (PAK1), and Src, to name only a few. A-kinase anchoring proteins (AKAPs) associate PKA with the actin cytoskeleton, thereby enhancing its signaling [73]. 

 It remains to be evaluated whether other kinases may phosphorylate IQGAP2 at different sites. Another recent work showed that Deleted in liver cancer 1 (DLC1), a Rho GTPase-activating protein, is a substrate for phosphorylation by Akt, and this phosphorylation negatively regulates the tumor suppressor function of DLC1 in liver [74]. Moreover, IQGAP1 was shown to coimmunoprecipitate with Akt in heart [75] and liver [44]. High homology and similar domain structure between IQGAP1 and IQGAP2 supports the notion that IQGAP2 might be an Akt substrate as well. Overactivation of the PI3K/Akt signaling pathway (evident by increased phosphorylation of both Akt and GSK3*β*) was observed in *Iqgap2* ^−/−^ livers [39]. This suggests that in IQGAP2-deficiency, overactivation of the PI3K/Akt signaling pathway may contribute to the development of HCC, along with the aberrantly activated Wnt/*β*-catenin pathway [27]. More direct evidence of IQGAP2 involvement in the PI3K signaling network has been provided as a result of solving the crystal structure of the IQGAP2 C-terminal domain [62]. It was shown that the extreme C-terminus of IQGAPs is responsible for binding to phosphatidylinositol 3,4,5-trisphosphate (PtdInsP_3_). Given that both IQGAP1 and IQGAP2 were reported to be able to bind PtdInsP_3_ with the similar affinity, further studies will be needed to distinguish the roles of the two IQGAPs in PI3K signaling. 

## 6. Concluding Remarks

 Mounting compelling evidence in support of IQGAP2 acting as a tumor suppressor in HCC and other cancers calls for further thorough studies of this intriguing protein. The fact that it counteracts oncogenic effects of its very close homolog, IQGAP1, makes these studies even more urgent and opens additional avenues for development of principally new therapeutics for HCC and perhaps other malignancies. Further research employing targeted functional proteomics will hopefully identify the whole spectrum of IQGAP2 binding partners and domains responsible for these interactions. Identification of downstream molecular effectors unique for each IQGAP would provide additional candidate targets. Likewise, means for specific blocking of IQGAP1 domains need to be explored, although this should be approached with caution. For instance, it was demonstrated that IQGAP1 is essential for the integrity of actin structures around bile canaliculi and inhibition of IQGAP1 would impact maintenance of stable adherens junctions in liver [76]. Finally, a better understanding of tissue-specific regulation of the IQGAP1 and IQGAP2 genes would provide additional options for urgently needed therapeutic intervention. Clearly, there are many gaps yet to be filled in our knowledge of IQGAPs.
